# Visible-Light-Driven CO Preferential Oxidation over In Situ Photodeposited Au/TiO_2_ Catalysts in H_2_-Rich Atmospheres

**DOI:** 10.3390/molecules31152560

**Published:** 2026-07-23

**Authors:** Qiuzhong Li, Renkun Huang, Lu Chen, Ruowen Liang, Guiyang Yan, Wenxin Dai

**Affiliations:** 1 College of New Energy and Materials, Ningde Normal University, Ningde 352100, China; t0319@ndnu.edu.cn (Q.L.); chenlu199104@163.com (L.C.); ygyfjnu@163.com (G.Y.); 2 Fujian Province University Key Laboratory of Green Energy and Environment Catalysis, Ningde Normal University, Ningde 352100, China; 3 Research Institute of Photocatalysis, State Key Laboratory of Photocatalysis on Energy and Environment, Fuzhou University, Fuzhou 350108, China; daiwenxin@fzu.edu.cn

**Keywords:** Au/TiO_2_, H_2_ rich, CO-PROX, visible light, in situ DRIFTS, in situ EPR

## Abstract

At low temperatures, the preferential removal of CO from the fuel feed of PEMFC is a critical factor for ensuring the optimal performance of fuel cells. In this study, Au/TiO_2_-PD and Au/TiO_2_-DP catalysts were synthesized via in situ photo-deposition and deposition–precipitation methods, respectively. The catalytic performance for CO preferential oxidation was evaluated in a hydrogen-rich atmosphere, and the effects of visible light irradiation on catalytic activity and selectivity were systematically investigated. The Au/TiO_2_-DP catalyst exhibited a relatively low CO conversion under dark conditions in the hydrogen-rich atmosphere, while visible light irradiation significantly enhanced its CO oxidation activity and selectivity. In contrast, the Au/TiO_2_-PD catalyst achieved a high CO oxidation conversion, but suffered from low CO oxidation selectivity; moreover, visible light exerted a weak inhibitory effect on its selectivity. Combined characterization results from temperature-programmed desorption (TPD), temperature-programmed surface reaction (TPSR), in situ diffuse reflectance infrared Fourier-transform spectroscopy (DRIFTS) and in situ electron paramagnetic resonance (EPR) revealed that the Au/TiO_2_-PD catalyst possessed stronger hydrogen adsorption, dissociation and oxidation capabilities than the Au/TiO_2_-DP catalyst. The rapid dissociation of hydrogen molecules over the Au/TiO_2_-PD catalyst accelerated the activation of adsorbed oxygen species and simultaneously promoted the formation of water via hydrogen oxidation. Excessive water accumulation on the catalyst surface occupied the active sites for CO oxidation, thereby imposing an overall inhibitory effect on CO preferential oxidation.

## 1. Introduction

The preferential oxidation of CO serves as a critical procedure for purifying the feed gas of proton exchange membrane fuel cells (PEMFCs) in hydrogen-rich gas mixtures [1]. Hydrogen generated via the water-gas shift reaction acts as the primary feed gas for PEMFCs, which inevitably contains a certain concentration of CO (0.5–2%). CO impurities can poison Pt electrodes [2], deactivate catalytic sites, and degrade the overall efficiency of fuel cells [3,4]. Therefore, the efficient and complete removal of CO at low temperatures in hydrogen-rich systems has long been a research hotspot with significant practical application value.

Supported gold nanoparticles (Au NPs) display outstanding low-temperature catalytic activity for CO oxidation and have been extensively investigated for CO preferential oxidation (CO-PROX) [5,6,7,8,9,10,11,12]. Previous work also confirms that H_2_ undergoes dissociation at low-coordination corner sites of Au NPs even at room temperature [13], and both the morphology and particle size of Au NPs strongly govern H_2_ dissociation efficiency [2]. In Au/TiO_2_ catalytic systems, H_2_ dissociates and adsorbs onto Au NP surfaces, then reacts with interfacial adsorbed oxygen to form surface-bound water. This adsorbed water competes with CO for active adsorption and oxidation sites [14]. At low H_2_ concentrations, less adsorbed water accumulates, facilitating selective CO oxidation. Mechanistically, surface water reacts with CO via intermediate -OOH species formed from activated O_2_ molecules to produce CO_2_; this pathway simultaneously boosts CO oxidation efficiency and enables cyclic regeneration of reaction-related water and catalyst active sites [11,15,16]. By contrast, elevated H_2_ concentrations intensify competitive adsorption and oxidation of H_2_ over Au particle surfaces, consuming substantial reactive oxygen species. This effect is markedly pronounced under high-H_2_ atmospheres and ultimately suppresses the overall CO oxidation performance.

In our previous work [17], we reported the synthesis and structural characterization of Au/TiO_2_-PD and Au/TiO_2_-DP catalysts, and systematically compared their catalytic performance toward visible-light-driven low-temperature CO oxidation. In this work, Au/TiO_2_-PD and Au/TiO_2_-DP catalysts were systematically investigated for their CO-PROX performance under H_2_-rich atmospheres. Combined characterizations including H_2_-TPD, TPSR, in situ DRIFTS, and in situ ESR were employed to compare the two catalysts with respect to differences in H_2_ adsorption, dissociation and oxidation, the capability of dissociated H species to activate oxygen, as well as the corresponding promotional and inhibitory effects on CO oxidation.

## 2. Results and Discussion

### 2.1. CO-PROX Catalytic Performance Under H_2_-Rich

**Figure 1:**
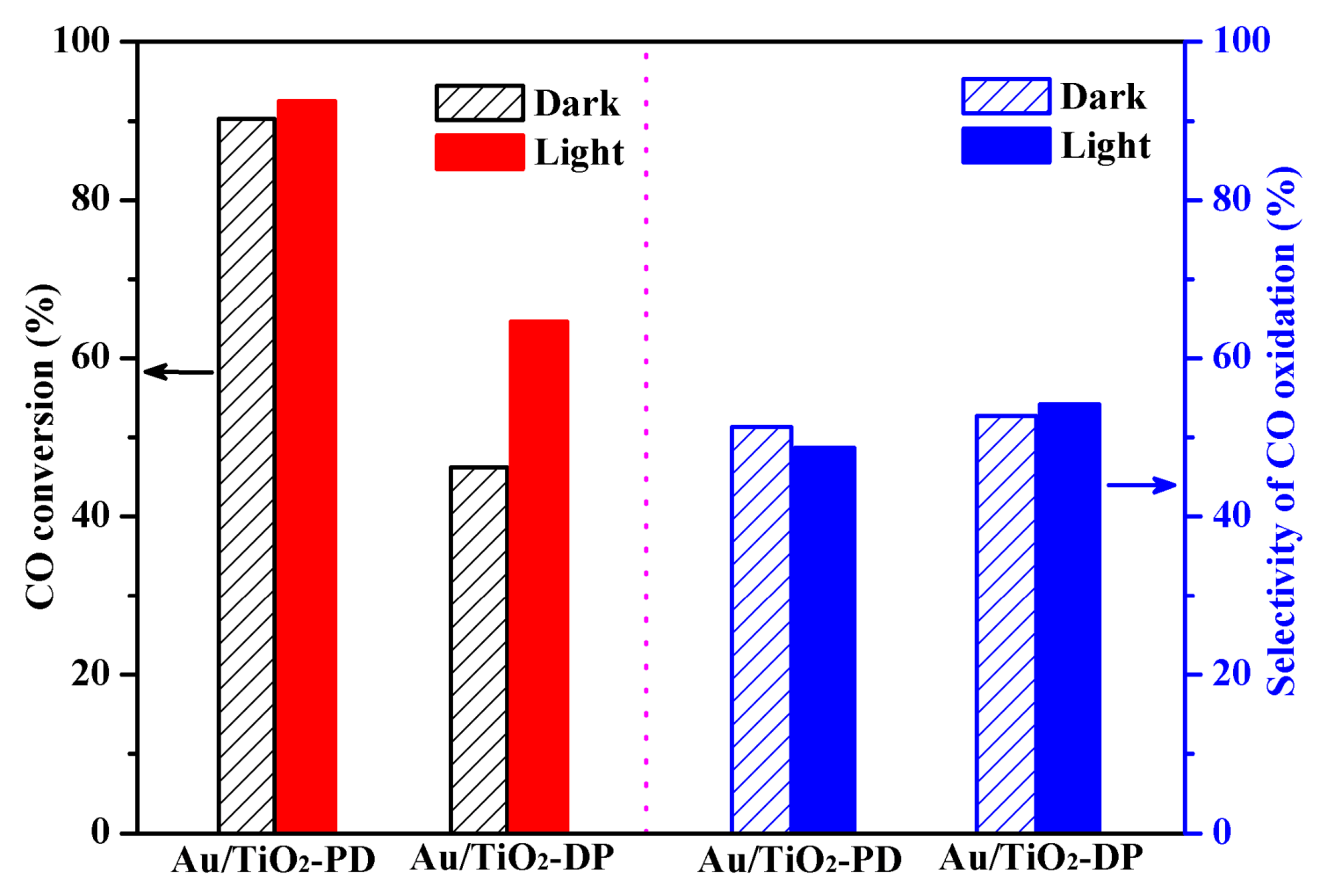
Conversion and selectivity of CO oxidation in H_2_-rich atmosphere over Au/TiO_2_-PD and Au/TiO_2_-DP catalysts under dark and visible light irradiation.

The CO oxidation conversion and selectivity of Au/TiO_2_-PD and Au/TiO_2_-DP catalysts under H_2_-rich conditions are presented in Figure 1. As shown in Figure 1, the Au/TiO_2_-PD catalyst achieved CO conversion values of 90.3% and 92.5% under dark and visible-light conditions, with corresponding CO-PROX selectivity of 51.3% and 48.7%. In comparison, the Au/TiO_2_-DP catalyst exhibited much lower CO conversion of 46.2% (dark) and 64.6% (visible light), while its selectivity remained relatively stable at 52.7% and 54.2%, respectively. These results indicate that Au/TiO_2_-PD delivers superior CO conversion efficiency yet slightly lower CO-PROX selectivity in a hydrogen-rich atmosphere. In contrast, Au/TiO_2_-DP presents a significantly inferior CO conversion performance under dark conditions. Upon visible-light irradiation, the CO conversion of Au/TiO_2_-PD was barely enhanced, whereas its selectivity declined, suggesting a negative light-induced inhibitory effect. Conversely, Au/TiO_2_-DP exhibited a prominent photo-promoting effect, with visible light simultaneously improving both its CO conversion and selectivity. Such distinct photocatalytic behaviors originate from the localized surface plasmon resonance (LSPR) effect of Au NPs. Visible light excitation increases the electron density of Au active sites, facilitates the adsorption and activation of O_2_ molecules, and further accelerates the generation of surface adsorbed water. This phenomenon is much more prominent on Au/TiO_2_-PD than on Au/TiO_2_-DP. Excessively generated surface water on Au/TiO_2_-PD occupies substantial active sites that would otherwise participate in CO oxidation, thereby suppressing CO-PROX selectivity. By contrast, Au/TiO_2_-DP possesses a weaker H_2_ dissociation capability, which effectively inhibits competitive H_2_ oxidation on the catalyst surface. This suppression of hydrogen side reactions moderately benefits CO oxidation performance. Nevertheless, the overall CO conversion and selectivity of the Au/TiO_2_-DP catalyst remain at a relatively low level.

### 2.2. Adsorption, Dissociation and Spillover Behaviors of H_2_

**Figure 2:**
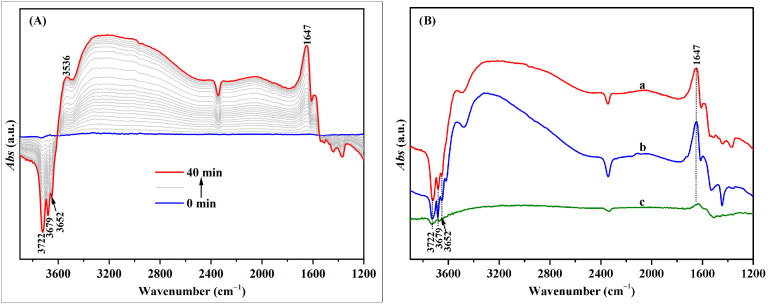
In situ DRIFTS spectra of H_2_ adsorption over (**A**) Au/TiO_2_-PD and (**B**) three different catalysts measured at room temperature under dark conditions. Curves a, b, and c correspond to Au/TiO_2_-PD, Au/TiO_2_-DP, and TiO_2_ after 40 min of H_2_ adsorption, respectively.

Panayotov et al. adopted in situ DRIFTS to investigate H_2_ dissociation on Au/TiO_2_ catalysts and the hydrogen spillover process onto the support surface [18]. Dissociated H atoms donate electrons to Au sites and are captured by the delocalized conduction band of TiO_2_, giving rise to broad infrared absorption bands in the mid-infrared region. Researchers commonly regard this conduction-band-enhanced broad IR absorption as direct evidence for H_2_ dissociation and spillover over Au NPs [19,20]. Herein, in situ DRIFTS measurements were performed to characterize H_2_ adsorption and dissociation on the as-prepared catalysts, and the corresponding spectra are presented in Figure 2. After H_2_ adsorption on Au/TiO_2_-PD at room temperature (Figure 2A), the intensity of the broad IR background absorption band within 2500–1800 cm^−1^ increased drastically, consistent with previously reported literature, confirming H_2_ adsorption and dissociation on the catalyst surface. The enhanced vibrational bands spanning 3600–2500 cm^−1^ correspond to the stretching vibrations of hydroxyl groups (*v*_OH_) originating from surface-adsorbed water [11]. Meanwhile, the bending vibration bands of adsorbed water (*δ*_HOH_) at 1647 cm^−1^ intensified, and three negative bands at 3722, 3679 and 3652 cm^−1^ were detected, assigned to Ti-OH vibrational bands at distinct surface sites [21]. The evolution of these negative bands demonstrates gradual consumption of surface hydroxyl groups, which further verifies that H atoms dissociated on Au sites spill over onto the TiO_2_ support and react with surface Ti-OH to form adsorbed water [22]. Figure 2B (curves b and c) displays the in situ DRIFTS spectra of H_2_ adsorption over bare TiO_2_ and Au/TiO_2_-DP, respectively. Bare TiO_2_ only exhibited a minor enhancement in broad background absorption, whereas Au/TiO_2_-DP showed a notable rise in IR absorption intensity, though this increment was far weaker than that observed for Au/TiO_2_-PD. In addition, the negative consumption bands of Ti-OH at 3722, 3679 and 3652 cm^−1^ were less intense for Au/TiO_2_-DP, demonstrating its inferior H_2_ adsorption and dissociation capacity.

**Figure 3:**
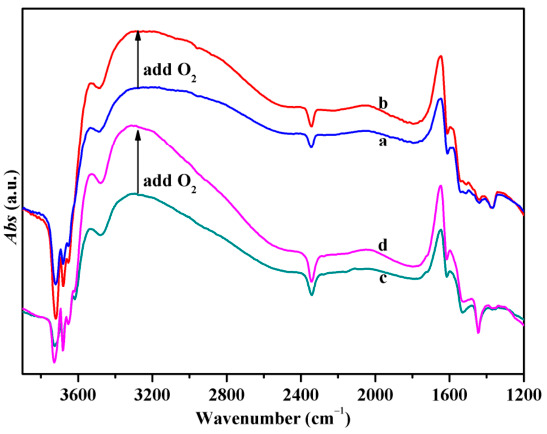
In situ DRIFTS spectra of Au/TiO_2_-PD and Au/TiO_2_-DP upon H_2_ adsorption followed by switching to an O_2_-He mixed gas atmosphere. Curves a and b represent the test results of the Au/TiO_2_-PD catalyst after H_2_ adsorption in the dark, followed by switching to an O_2_-He mixed gas atmosphere, respectively. Curves c and d correspond to the test results of the Au/TiO_2_-DP catalyst under the same dark-condition H_2_ adsorption and subsequent O_2_-He mixed gas switching process.

Subsequent experiments were conducted by introducing O_2_-He mixed gas after saturating Au/TiO_2_-PD and Au/TiO_2_-DP with pre-adsorbed H_2_, with the resultant spectra summarized in Figure 3. Following O_2_ introduction, the vibrational bands of surface-adsorbed water at 1647 cm^−1^ and 3600–2500 cm^−1^ increased dramatically. For Au/TiO_2_-PD (curves a and b), the Ti-OH vibrational signals at 3722, 3679 and 3652 cm^−1^ decreased substantially again, revealing that hydroxyl groups generated via H spillover onto TiO_2_ were further oxidized to additional adsorbed water. By contrast, only slight attenuation of Ti-OH signals was observed for Au/TiO_2_-DP (curves c and d). These results prove that adsorbed O_2_ molecules preferentially react with dissociated H atoms to produce surface-bound water after oxygen exposure, which is the dominant surface reaction pathway.

**Figure 4:**
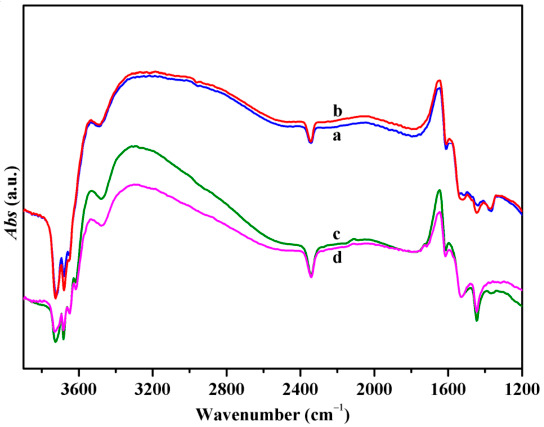
In situ DRIFTS spectra for H_2_ adsorption over Au/TiO_2_-PD and Au/TiO_2_-DP catalysts at room temperature with and without visible-light irradiation. Curves a and b correspond to H_2_ adsorption on Au/TiO_2_-PD under dark conditions and visible-light irradiation, respectively; curves c and d represent H_2_ adsorption on Au/TiO_2_-DP under dark conditions and visible-light irradiation, respectively.

We further characterized H_2_ adsorption behaviors of Au/TiO_2_-PD and Au/TiO_2_-DP under dark and visible-light conditions, and the relevant spectra are shown in Figure 4. Upon visible-light irradiation, the broad IR background absorption of Au/TiO_2_-PD increased marginally, indicating that visible light barely facilitates H_2_ dissociation on Au/TiO_2_-PD. However, Au/TiO_2_-DP exhibited distinct spectral variations under visible light: the vibrational intensity of surface-adsorbed water declined, accompanied by weakened *δ*_HOH_ signals. This observation indicates that visible light inhibits H_2_ dissociation and spillover over Au/TiO_2_-DP, which explains its improved CO-PROX selectivity under illumination.

**Figure 5:**
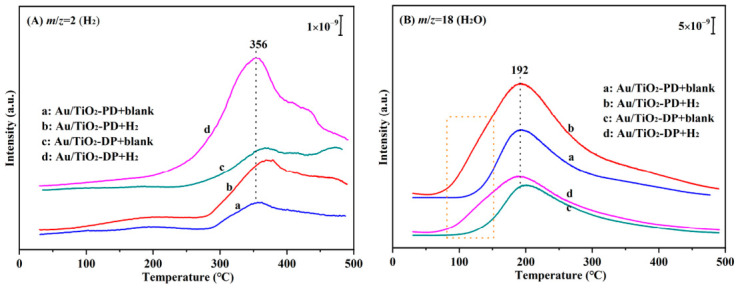
Mass spectral signals of (**A**) H_2_ and (**B**) H_2_O desorption during TPD measurements following H_2_ adsorption on Au/TiO_2_-PD and Au/TiO_2_-DP catalysts, respectively.

To further distinguish the H_2_ adsorption and dissociation performances of Au/TiO_2_-PD and Au/TiO_2_-DP, Figure 5 displays mass spectral signals of H_2_ (*m*/*z* = 2) and H_2_O (*m*/*z* = 18) recorded during H_2_-TPD measurements. Pure He was used for blank TPD tests to provide reference background signals. As shown in Figure 5, both catalysts exhibited a water desorption peak near 192 °C (Figure 5B) and an H_2_ desorption peak at approximately 356 °C (Figure 5A), originating from desorption of surface chemically bound water and hydroxyl species. After H_2_ pre-adsorption, H_2_ desorption signals began to rise at ~200 °C, suggesting the onset of H_2_ recombination. Moreover, the H_2_ desorption peak at 356 °C was markedly intensified relative to the blank spectrum. Correspondingly, the half-peak width of the water desorption signal at 192 °C broadened with elevated peak intensity, and water desorption was detected starting at 80 °C (dashed box in Figure 5B). This confirms that dissociated hydrogen undergoes oxidation to form surface-adsorbed water, which desorbs at relatively low temperatures. Comparative analysis of peak intensities reveals that Au/TiO_2_-DP only showed a slight rise in water desorption signals but a prominent enhancement of H_2_ desorption peaks. This implies that dissociated H atoms on Au/TiO_2_-DP tend to recombine into gaseous H_2_, and partial surface water decomposes to release H_2_. In contrast, Au/TiO_2_-PD presented a mild increase in H_2_ desorption signals yet a dramatic boost in water desorption intensity. This indicates that adsorbed hydrogen on Au/TiO_2_-PD is readily oxidized and desorbed in the form of H_2_O. Collectively, H_2_ dissociation and subsequent oxidation proceed much more readily on Au/TiO_2_-PD than Au/TiO_2_-DP, which accounts for the poor CO-PROX selectivity of Au/TiO_2_-PD under H_2_-rich atmospheres.

### 2.3. Impacts of H_2_ Adsorption and Dissociation on Oxygen

**Figure 6:**
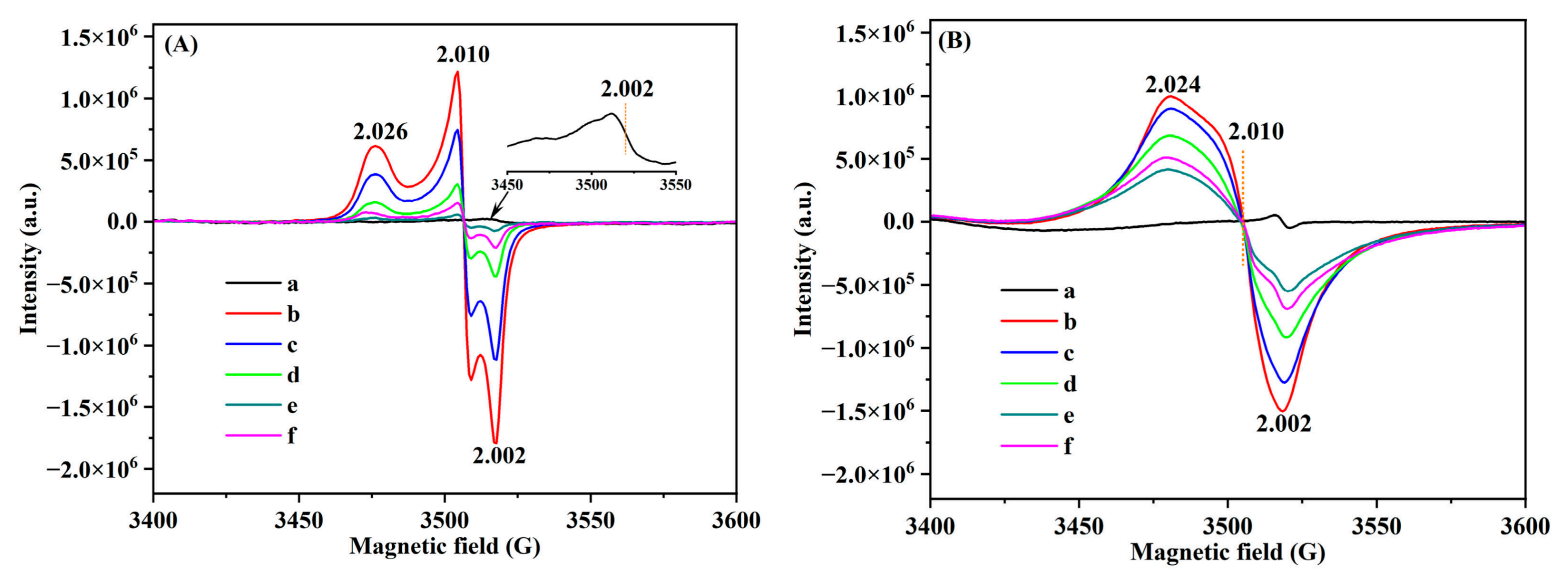
In situ EPR spectra collected at room temperature for (**A**) Au/TiO_2_-PD and (**B**) Au/TiO_2_-DP catalysts: (a) after room-temperature evacuation; (b–e) upon exposure to 20 μmol H_2_ for 2, 4, 10, and 20 min, respectively; (f) after H_2_ exposure followed by 2 min of visible-light irradiation.

At room temperature, H_2_ can adsorb and dissociate on Au NPs, competing with CO for active adsorption and oxidation sites and thus altering CO-PROX selectivity. To clarify the differences in H_2_ adsorption and dissociation between Au/TiO_2_-PD and Au/TiO_2_-DP, in situ EPR measurements were carried out for H_2_-adsorbed catalyst samples. Prior to testing, samples were evacuated at room temperature to eliminate atmospheric interference. EPR spectra collected before and after H_2_ adsorption, as well as under visible-light irradiation, are presented in Figure 6. After vacuum degassing, Au/TiO_2_-PD exhibited a weak oxygen vacancy signal at *g* = 2.002, assigned to chemically adsorbed oxygen species ($O_{2}^{-}$). Upon H_2_ introduction, the EPR signal at *g* = 2.002 intensified rapidly, accompanied by obvious signals at *g* = 2.010 and 2.026 corresponding to Ti^4+^ rearrangement peaks [23]. As testing proceeded, the Ti^4+^-$O_{2}^{-}$ related peak intensities decayed rapidly and were nearly fully consumed after 20 min of H_2_ exposure. The evacuated Au/TiO_2_-DP sample displayed a distinct oxygen vacancy signal. After H_2_ adsorption, similar Ti^4+^ rearrangement peaks at *g* = 2.010 and 2.024 were observed. Nevertheless, its signal evolution differed greatly from Au/TiO_2_-PD: noticeable Ti^4+^ rearrangement peaks remained detectable even after 20 min of H_2_ treatment. EPR results demonstrate that after H_2_ dissociation on Au NPs, electrons transfer onto Au sites and rapidly migrate to the support surface, reducing O_2_ adsorbed at defect sites into superoxide species ($O_{2}^{-}$). Dissociated H atoms spill over onto the support at a low migration rate and combine with $O_{2}^{-}$ to form ·OOH or ·OH intermediates, which are ultimately converted into surface-adsorbed water on Ti^4+^ sites and gradually attenuate the $O_{2}^{-}$ signals [20]. Comparison of EPR signal variations confirms that Au/TiO_2_-PD possesses superior H_2_ adsorption, dissociation and hydrogen oxidation capacity relative to Au/TiO_2_-DP, consistent with its lower CO-PROX selectivity. This trend aligns with catalytic performance and in situ DRIFTS results. Additionally, visible-light irradiation strengthened the Ti^4+^ rearrangement peaks, further verifying that the LSPR effect of Au NPs elevates surface electron density and facilitates oxygen activation.

### 2.4. Promotion and Inhibition Effects of H_2_ on CO Oxidation

In situ DRIFTS characterizations confirm that H atoms dissociated on Au NPs spill over onto TiO_2_ to generate surface-adsorbed water. This water participates in CO oxidation to produce CO_2_ while regenerating surface hydroxyl groups, enabling cyclic recovery of catalyst active sites [9]. Numerous studies have explored how surface-adsorbed H_2_O and -OH influence CO oxidation over Au-based catalysts, concluding that moderate amounts of surface water can boost CO catalytic activity [24]. Thompson’s group systematically investigated CO oxidation over Au/MgO and first proposed the mechanism involving surface hydroxyl participation. Their core conclusion states that CO adsorbed on Au sites reacts with bound hydroxyls (Au^δ+^-OH) to form carbonate intermediates; meanwhile, O_2_ molecules adsorb and activate at support oxygen vacancies to generate reactive oxygen species, which react with carbonates to release CO_2_. Hydroxyl groups consumed during the reaction can be continuously regenerated [25]. Daté et al. further reported that surface hydroxyls accelerate CO oxidation by activating O_2_ or promoting the decomposition of carbonate intermediates [26].

**Figure 7:**
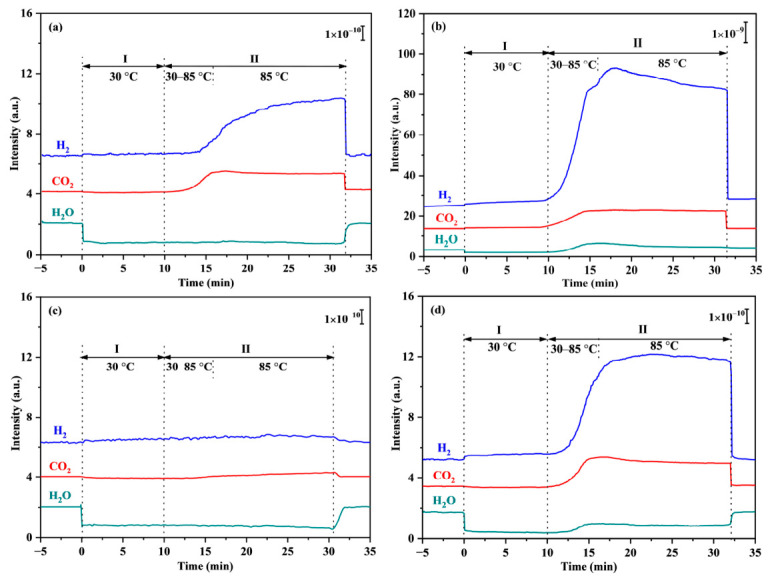
Mass spectral profiles recorded during TPSR tests over (**a**,**b**) Au/TiO_2_-PD and (**c**,**d**) Au/TiO_2_-DP catalysts with and without pre-adsorbed H_2_. Curves a and c correspond to samples without pre-adsorbed H_2_, while curves b and d represent samples with pre-adsorbed H_2_, respectively.

To fully uncover how pre-adsorbed hydrogen regulates CO-PROX performance, TPSR measurements were performed on samples with and without H_2_ pre-adsorption. H_2_-TPD results reveal that intrinsic surface water and hydroxyl groups on the catalyst desorb below 200 °C. To exclude interference from native surface water and hydroxyls, all samples were pretreated at 200 °C for 1 h under high-purity He flow. Trace CO_2_, H_2_ and H_2_O exist in both CO-He reaction gas and He carrier gas with different concentrations, leading to abrupt mass spectral signal fluctuations during gas switching. Therefore, the initial mass spectral signal recorded after introducing reaction gas was set as the baseline, and signal deviations from this baseline were used to quantify the formation or consumption of surface species. TPSR spectra with and without H_2_ pre-adsorption are shown in Figure 7, and corresponding mass spectral variations in CO_2_, H_2_ and H_2_O are summarized in Table 1. Without H_2_ pre-adsorption, Au/TiO_2_-PD generated negligible CO_2_ in the initial heating stage, accompanied by slight H_2_ desorption and minor water consumption. In this stage, CO interacts with surface active species to form reaction intermediates. Obvious CO_2_ production was observed in the subsequent heating and isothermal stages, alongside enhanced H_2_ desorption and further water consumption, which is attributed to the decomposition of surface carbonate intermediates and the water-gas shift reaction. After H_2_ pre-adsorption, CO oxidation was drastically promoted, with sharply elevated mass spectral signals for CO_2_, H_2_ and H_2_O. Specifically, the CO_2_ signal intensity increased sixfold compared with the H_2_-free test, while the H_2_ signal rose by 19 times, accompanied by abundant desorption of surface water. For Au/TiO_2_-DP, only trace CO_2_ and H_2_ were detected during heating without H_2_ pre-adsorption. Even after H_2_ pre-adsorption, the desorption yields of CO_2_, H_2_ and H_2_O were far lower than those measured for Au/TiO_2_-PD. These findings verify that Au/TiO_2_-PD exhibits stronger H_2_ dissociation and oxidation capacity, and trace water generated from hydrogen oxidation delivers a favorable promoting effect on CO oxidation.

**Figure 8:**
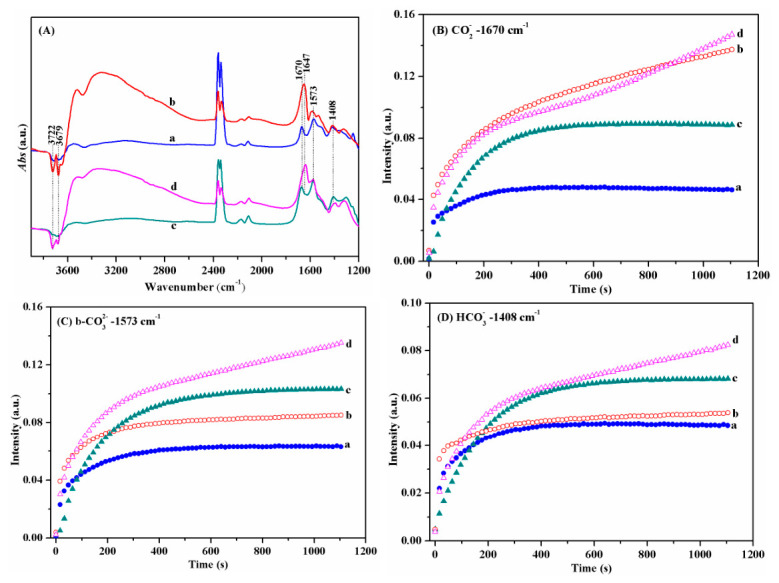
(**A**) in situ DRIFTS spectra of (a, b) Au/TiO_2_-PD and (c, d) Au/TiO_2_-DP catalysts measured under H_2_-free and H_2_-rich atmospheres, respectively. (**B**–**D**) Time-dependent intensity profiles of infrared adsorption bands assigned to reaction intermediates. Curves a and c correspond to the H_2_-free condition, while curves b and d represent the H_2_-rich atmosphere, respectively.

Under H_2_-rich conditions, competitive adsorption between H_2_ and CO on Au active sites suppresses CO-PROX activity, as further validated by in situ DRIFTS. Figure 8 presents in situ DRIFTS spectra of CO/O_2_ mixtures reacting over Au/TiO_2_-PD and Au/TiO_2_-DP under H_2_-free and H_2_-rich atmospheres, together with time-resolved intensity curves of characteristic IR bands corresponding to key CO oxidation intermediates. In the absence of hydrogen, intense CO_2_ absorption bands were detected, indicating rapid CO oxidation into CO_2_. Upon introducing excess H_2_, CO_2_ signal intensity dropped significantly, reflecting severe inhibition of CO oxidation. Meanwhile, intensified broad background absorption, consumption of surface Ti-OH, and enhanced vibrational signals of adsorbed H_2_O were observed. The intensity of intermediate-related IR bands reflects the accumulation degree of surface intermediates: stronger signals correspond to greater intermediate accumulation, which hinders CO conversion. Under H_2_-free conditions, intermediate signals of both catalysts remained weak and stable over time. In H_2_-rich environments, intermediate signal intensities increased for both samples, with a dramatic rise in carboxylate signals for Au/TiO_2_-PD (Figure 8B). This proves that H_2_ readily dissociates on Au/TiO_2_-PD and reacts with activated oxygen to form Au-OOH species, which further react with CO adsorbed on Au sites to generate Au-COOH intermediates. The above results collectively demonstrate that under H_2_-rich conditions, Au/TiO_2_-PD not only enables efficient H_2_ adsorption and dissociation but also hinders the transformation of CO oxidation intermediates. Accumulated surface intermediates block active site regeneration and ultimately restrain CO oxidation [27].

In summary, the Au/TiO_2_-PD catalyst displays markedly enhanced H_2_ adsorption and dissociation relative to Au/TiO_2_-DP. Benefiting from the visible-light-induced LSPR effect, the surface charge density of Au/TiO_2_-PD is further boosted, facilitating the activation and cleavage of molecular oxygen [17]. The activated oxygen species readily react with spillover H to generate adsorbed water, which occupies accessible active sites. This process also promotes the buildup of carboxylate intermediates on the catalyst surface, substantially suppressing CO adsorption and subsequent oxidation. By contrast, Au/TiO_2_-DP affords inferior CO conversion yet possesses weaker capacity for H_2_ adsorption, dissociation and oxidation. Additionally, visible light imposes a moderate inhibitory influence on its catalytic activity, which rationalizes its higher selectivity toward the target reaction.

### 2.5. Dissociative, Spillover and Oxidation Mechanism of H_2_

Au NPs possess low H_2_ dissociation energy, and hydrogen oxidation proceeds via the Langmuir–Hinshelwood (L-H) mechanism. DFT calculations reported by Green et al. revealed that H_2_ dissociation energy reaches ~0.5 eV without oxygen, which decreases to 0.22 eV in oxygen-containing environments [20,28]. The authors proposed that hydrogen oxidation occurs at the dual Au-TiO_2_ interfacial sites: H_2_ molecules adsorb and dissociate on Au, and one H atom spills over to adjacent interfacial sites to react with bridge-bonded O_2_ on five-coordinated Ti (Ti_5_c), forming Ti-OOH (Step 1). The O-O bond in Ti-OOH cleaves to generate Ti-OH and Ti-O (Step 2). Continuously spilled H atoms further react with Ti-OH and Ti-O to produce surface-adsorbed water on Ti_5_c sites (Steps 3 and 4). Interfacial water molecules subsequently desorb as free water (Step 5), a process requiring high desorption energy. The detailed reaction steps are listed as follows:

(1) $${\text{Au-H}}_{2}+{\text{Ti-O}}_{2}^{-}⟶\text{Au-H}+\text{Ti-OOH}$$

(2) Ti-OOH⟶Ti-O+Ti-OH

(3) Ti-O+Au-H⟶Ti-OH

(4) $$\text{Ti-OH}+\text{Au-H}⟶{\text{Ti-OH}}_{2}$$

(5) $${\text{Ti-OH}}_{2}⟶{\mathrm{Ti}+H}_{2}O$$

### 2.6. Promotion and Inhibition Mechanism of H_2_ on CO Oxidation

Trace water existing in the reaction system facilitates CO oxidation over Au/TiO_2_ catalysts [11,15,27,29,30,31,32]. Water generated from H_2_ dissociation and oxidation at the Au-TiO_2_ interface either adsorbs stably on Ti_5_c sites or interacts with surface hydroxyl groups via hydrogen bonds after partial desorption. O_2_ molecules adsorb and activate at Au-TiO_2_ interfaces and combine with protons donated by surface water to form Au-OOH intermediates (Step 6). These ·OOH species participate in CO oxidation (Steps 7 and 8), regenerating water molecules in Step 8 and completing a catalytic cycle [30]:

(6) $${\mathrm{Au}}^{\delta –}{\;+\;O}_{2}{\;+\;\text{Ti-OH}}_{2}\to \text{Au-OOH}\;+\;\text{Ti-OH}$$

(7) $${\text{Au-OOH}+\mathrm{Au}}^{\delta –}\text{-}\mathrm{CO}\to \text{Au-COOH}+\text{Au-O}$$

(8) $$\text{Au-COOH}\to {\mathrm{CO}}_{2}+\text{Au-H}$$

(9) $$\text{Au-H}+\text{Ti-OH}\to {\mathrm{Au}}^{\delta –}{+\text{Ti-OH}}_{2}$$

Accordingly, trace hydrogen in the feed generates moderate surface water that positively accelerates CO oxidation. However, excess H_2_ introduces two inhibitory effects under H_2_-rich conditions: first, dissociated H atoms compete fiercely with CO for Au active sites; second, massive amounts of water accumulate on the catalyst surface [32]. Given the high desorption barrier of water on Ti_5_c, water molecules persistently occupy Au-TiO_2_ interfacial and surface active sites, reduce CO turnover frequency, and present an overall inhibitory effect on CO oxidation.

## 3. Materials and Methods

### 3.1. Preparation of Catalysts

Referring to our previous work [17], Au/TiO_2_-PD and Au/TiO_2_-DP catalysts were fabricated via the in situ photodeposition (PD) and deposition–precipitation (DP) routes, respectively. The detailed synthetic procedure for Au/TiO_2_-PD is provided below. A volume of 1.2 mL HAuCl_4_ aqueous solution (0.01 g·mL^−1^) was diluted with 100 mL deionized water, and the pH of the mixed solution was tuned to 9 using 0.1 mol·L^−1^ NaOH solution. Subsequently, 1.0 g TiO_2_(EG) powder was accurately weighed and dispersed into the above mixture under continuous stirring for 2 h to form a homogeneous suspension. The suspension was then subjected to 10 min irradiation using a PLS-SXE300 xenon lamp (Beijing Perfectlight Technology Co., Ltd., Beijing, China) fitted with a 365 nm band-pass filter. The purple precipitate was harvested by centrifugation and thoroughly washed with ultrapure water multiple times. After drying in a blast drying oven at 80 °C for 12 h, the solid precursor was calcined at 350 °C for 3 h with a temperature ramp rate of 1 °C·min^−1^ starting from room temperature. The synthetic protocol for Au/TiO_2_-DP is outlined as follows. Briefly, 1.2 mL of 0.01 g·mL^−1^ HAuCl_4_ aqueous solution was mixed with 100 mL deionized water, and the pH value was adjusted to 9 with 0.1 mol·L^−1^ NaOH solution. A total of 1.0 g TiO_2_ powder was added to the suspension and magnetically stirred for 2 h under dark conditions. Afterwards, 0.1 mol·L^−1^ NaBH_4_ aqueous solution was added for 2 h of reduction treatment. The resulting precipitate was collected by centrifugation, rinsed repeatedly with deionized water, and finally dried at 80 °C for 12 h.

### 3.2. Catalyst Characterizations

In situ EPR spectra were acquired on a Bruker (Billerica, MA, USA) A-300E spectrometer at room temperature, with an operating frequency of 9.84 GHz and microwave power of 15.92 mW.

In situ DRIFTS measurements were performed on a Bruker (Billerica, MA, USA) Vertex 80v FT-IR spectrometer equipped with an MCT detector, coupled with a custom-built in situ DRIFTS reactor cell and a smart collector accessory. Before testing, the sample was loaded into the DRIFTS reactor cell and pretreated by evacuation at 200 °C for 1 h under a He atmosphere (flow rate: 50 mL·min^−1^) to eliminate physically adsorbed water. After cooling naturally to 25 °C, the background spectrum of the sample was recorded. Subsequently, a probe gas mixture consisting of 5% H_2_ balanced with high-purity N_2_ was introduced into the reaction system at a constant total flow rate of 10 mL·min^−1^. Spectral data were continuously collected for 40 min under dark and visible light conditions, respectively. Thereafter, the feed gas was switched to 5% O_2_ balanced with high-purity He while maintaining the same total flow rate of 10 mL·min^−1^. All in situ DRIFTS spectra were recorded over a wavenumber range of 4000–1000 cm^−1^ with a spectral resolution of 8 cm^−1^. Each spectrum was acquired by averaging 64 scans to improve the signal-to-noise ratio.

TPD and TPSR were carried out with a Micromeritics (Norcross, GA, USA) Autochem2910 instrument. In the TPD experiments, a specific process was used for the samples, as in the previous work of our research group [33]. The TPSR experiments for the samples proceeded as follows: (1) Prior to the introduction of adsorbed gas, a 100 mg sample was first pre-heat treated in high-purity He gas (50 mL·min^−1^) at 200 °C for 1 h. (2) After cooling down to 30 °C, the pre-adsorbed stream of 10% H_2_-Ar (50 mL·min^−1^) mixture gas was introduced into the sample for 30 min at room temperature. (3) Switch to He gas for 10 min. (4) Let us switch to the reaction gas (5% CO-He, 30 mL·min^−1^) and keep it at room temperature for 10 min, at the same time recording the signal of TCD, and the mass signals of *m*/*z* = 2, 18, and 44 were recorded to detect the masses of H_2_, H_2_O and CO_2_ molecules, respectively. (5) Finally, heat to 85 °C at a ramping rate of 10 °C·min^−1^, and hold for 20 min.

### 3.3. Catalytic Performances

CO preferential oxidation catalytic performance under H_2_-rich atmospheres was evaluated in an atmospheric-pressure fixed-bed flow reactor [34]. A total of 300 mg of catalyst with particle sizes ranging from 0.2 to 0.3 mm was loaded into a square quartz reactor cell fitted with a circulating cooling water system to maintain the reaction temperature at 25 °C, with a total gas flow rate of 100 mL·min^−1^. The reaction feed gas consisted of 0.3% CO, 0.3% O_2_, 50% H_2_, and high-purity He as the balance carrier gas for H_2_-rich PROX tests. Visible light irradiation was supplied by a PLS-SXE300 xenon lamp (Beijing Perfectlight Technology Co., Ltd., Beijing, China) equipped with two optical cutoff filters: one for blocking ultraviolet light (λ ≤ 420 nm) and another for filtering infrared radiation (λ > 760 nm). For dark-condition catalytic measurements, the quartz cell was fully wrapped with aluminum foil to eliminate all light illumination. Effluent gas compositions were continuously analyzed online via an Agilent 7890D (Santa Clara, CA, USA) gas chromatograph (GC) outfitted with a thermal conductivity detector (TCD) and a TDX-01 packed column (Santa Clara, CA, USA).

The CO conversion (*X*_CO_, %) and CO selectivity (*S*, %) in rich H_2_ were calculated as follows:

$$X_{\mathrm{CO}}(\%)\;=\left\{\frac{{\left[\mathrm{CO}\right]}_{\mathrm{in}}-{\left[\mathrm{CO}\right]}_{\mathrm{out}}}{{\left[\mathrm{CO}\right]}_{\mathrm{in}}}\right\}\times \;100\%\;$$

$$S\;(\%)=\left\{\frac{1}{2}\cdot \frac{{\left[\mathrm{CO}\right]}_{\mathrm{in}}-{\left[\mathrm{CO}\right]}_{\mathrm{out}}}{{{[O}_{2}]}_{\mathrm{in}}-{{[O}_{2}]}_{\mathrm{out}}}\right\}\times \;100\%\;$$

## 4. Conclusions

For CO preferential oxidation under H_2_-rich atmospheres, the in situ photodeposited Au/TiO_2_-PD catalyst achieves high CO conversion but suffers from low CO-PROX selectivity. Visible-light irradiation barely improves CO conversion and imposes a photo-induced inhibitory effect on selectivity. In contrast, Au/TiO_2_-DP delivers low intrinsic CO conversion but exhibits a prominent photo-promoting effect that enhances CO-PROX selectivity under illumination. Compared with Au/TiO_2_-DP, Au/TiO_2_-PD exhibits remarkably stronger H_2_ adsorption, dissociation and oxidation capacity. Dissociated hydrogen not only promotes oxygen activation but also accelerates surface hydrogen oxidation over Au/TiO_2_-PD. Trace surface water benefits CO oxidation activity, yet excessive water generated from massive hydrogen oxidation under H_2_-rich conditions covers active sites with low desorption capacity, aggravates the accumulation of CO oxidation intermediates, and ultimately suppresses CO conversion efficiency.

## Figures and Tables

**Table 1:** Variations in mass spectral signals of CO_2_, H_2_, and H_2_O recorded during TPSR measurements over Au/TiO_2_-PD and Au/TiO_2_-DP catalysts.

Pre-Adsorption of H_2_	Processes	Variations in Mass Signals, a.u. (10^−10^) *					
		Au/TiO_2_-PD			Au/TiO_2_-DP		
		CO_2_	H_2_	H_2_O	CO_2_	H_2_	H_2_O
Without	I	0	0.1	−0.1	−0.1	0.2	0
	II	1.4	3.7	−0.2	0.3	0.3	−0.2
With	I	0.2	1.6	0.1	0	0.2	−0.1
	II	8.4	63.8	4.4	2.0	6.7	0.5

*—The variation in each mass signal is defined as the signal intensity recorded during the reaction process minus the baseline intensity of pure CO flowing over Au/TiO_2_-PD and Au/TiO_2_-DP catalysts. Here, the mass signal is a value without units, and 10^−10^ is another expression of 10^−10^.

## Data Availability

Data are contained within the article.
